# Graphene Nanoplatelet Distribution Governs Thermal Conductivity and Stability of Paraffin-Based PCMs

**DOI:** 10.3390/nano15080587

**Published:** 2025-04-11

**Authors:** Levina E. A. Wijkhuijs, Pauline Schmit, Ingeborg Schreur-Piet, Henk Huinink, Remco Tuinier, Heiner Friedrich

**Affiliations:** 1 Laboratory of Physical Chemistry, Department of Chemical Engineering and Chemistry, Eindhoven University of Technology, P.O. Box 513, 5600 MB Eindhoven, The Netherlands; 2 Institute for Complex Molecular Systems, Department of Chemical Engineering and Chemistry, Eindhoven University of Technology, P.O. Box 513, 5600 MB Eindhoven, The Netherlands; 3 Eindhoven Institute for Renewable Energy Systems (EIRES), Eindhoven University of Technology, P.O. Box 513, 5600 MB Eindhoven, The Netherlands; 4 Center for Multiscale Electron Microscopy (CMEM), Department of Chemical Engineering and Chemistry, Eindhoven University of Technology, P.O. Box 513, 5600 MB Eindhoven, The Netherlands; 5 Transport in Permeable Media, Department of Applied Physics and Education, Eindhoven University of Technology, P.O. Box 513, 5600 MB Eindhoven, The Netherlands

**Keywords:** graphene nanoplatelets, thermal conductivity, paraffin PCM, filler network, shape stability, percolation

## Abstract

Materials for heat storage are important to fully utilize renewable energy sources and to realize a constant, on-demand supply. Organic phase change materials (PCMs) can play a crucial role in heat storage, as they have many advantages; however, their widespread commercial adoption is hindered by their low thermal conductivity and lack of cyclic stability. To enhance performance, highly thermally conductive fillers such as graphene nanoplatelets (GNPs) have been used; however, the role of the filler network has not been investigated. Here, we present, from a colloidal perspective, an in-depth study of GNP networks in paraffin PCMs. We investigate how GNP size, aspect ratio, and network topology determine thermal conductivity and cyclic stability of the composite. Our results show that the best-performing GNP network is random, with an optimized GNP aspect ratio. Filler fractions should be such that overlap between GNPs is guaranteed, which prevents leakage of paraffin from the composite, ensuring cyclic stability. These results not only contribute valuable insights into the design of new PCM composites but also emphasize the significance of considering filler geometry and network topology alongside filler type and fraction for optimizing thermal performance and cyclic stability.

## 1. Introduction

Renewable energy sources are key to realizing intergenerational equity in energy supply. To this end, energy storage solutions are urgently needed that can overcome the intermittent supply of renewable energy. Importantly, over 75 % of the domestic demand is related to thermal energy, such as space heating, water heating, and cooking [1]. To supply this demand, the use of thermal energy storage (TES) systems seems a convenient and efficient way [2,3]. TES can be divided into sensible heat storage (SHS), latent heat storage (LHS), and thermo-chemical heat storage (THS). Of these three approaches, LHS using phase change materials (PCMs) is a promising solution due to their variable energy storage duration (hours to days), high energy density (190–260 kJ/kg) [4], and tunable operating temperature (*T*_m_) [4,5,6]. PCMs have been widely studied in the fields of building science [7,8,9], thermal management [10,11], and solar energy [12]. The interest in PCMs is mainly because of the wide range of available PCM materials and almost isothermal operation of thermal storage and retrieval [13,14]. To develop desired PCMs for domestic energy storage, the thermophysical, chemical and economic factors need to be optimized. Viable PCMs:

(a) Have a relatively short charge and discharge time, in the order of minutes [15,16]. This means that a sufficiently high thermal conductivity in the solid as well as in the liquid phase needs to be achieved.
(b) Can store large amounts of energy and thus have a high latent heat (>200 kJ/kg).
(c) Exhibit a stable and well-defined phase transition at the operation temperature, i.e., a congruent phase change with little supercooling.
(d) Have a long lifetime (10 years or >3650 cycles). This means that the material should be thermally and chemically stable during repeated cycling and non-corrosive.
(e) For commercialization, the synthesis/preparation process needs to be industrially scalable, and materials need to be available on a large scale at low cost.

PCMs can be classified into organic, inorganic, and binary eutectic^1^ mixtures (Table 1). Inorganic PCMs have a high specific latent heat (100–200 kJ/kg) and a relatively high thermal conductivity (0.5–1.5 W m^−1^·K^−1^) compared to organic PCMs [4,20,21,22,23]; however, they suffer from supercooling, lack of thermal stability, chemical instability, and phase decomposition [24,25]. Salt hydrates, as an example of inorganic PCMs, suffer from metastability, a hysteresis between hydration and dehydration [26], resulting in density differences and phase segregation. Furthermore, it is difficult to tune their operating temperature (*T_m_*). Binary eutectic PCMs, on the other hand, show no phase segregation during thermal cycling [27,28]. Another advantage of binary eutectic PCMs is their relatively high specific latent heat [29,30,31]. However, they also have the disadvantage that their melting temperature cannot easily be tuned since it is determined by the type of binary eutectic mixture used [32]. A change in operating temperature would require the use of a different binary eutectic combination [20].

Organic PCMs overcome most of the problems mentioned, since they are chemically stable, non-corrosive, have a good latent heat of fusion (130–260 kJ/kg) [4,20,33], and have an easily adaptable operating temperature tunable by increasing or decreasing the molecules chain length [34,35]. This adaptability can be seen in the wide variety of organic PCM applications in the medical field, such as neonatal cooling [36], cold storage for medication [37], thermal therapy [38] and medical dressings [39]. The main disadvantages of organic PCMs, however, is their flammability, low thermal conductivity, and lack of shape stability during thermal cycling [40]. Due to the many advantages, and since disadvantages can be overcome by adding highly thermally conductive fillers, we will focus in this research on organic PCMs, i.e., paraffin.

To increase the thermal conductivity of organic PCMs, highly thermally conductive fillers may be added, such as metal or metal oxide nanoparticles [41,42], metal matrix [43,44] or carbon-based materials [45,46,47]. Common carbon-based materials, such as graphene, graphite, graphene nanoplatelets (GNPs), expanded graphite (EG) and carbon nanotubes (CNTs) are suitable fillers [46,48] due to their low thermal expansion coefficient, high chemical stability, and high thermal conductivity (graphene: ~5000 W·m^−1^·K^−1^, GNP: ~2000 W·m^−1^·K^−1^, CNT: ~3500 W·m^−1^·K^−1^) [49,50,51,52]. The high in-plane thermal conductivity of these carbon materials arises from the strong sp^2^ bonds between carbon atoms, allowing phonons to travel at high velocities through the strong anisotropic bonds of the carbon material [53,54].

It has been observed that the thermal conductivity of phase change composites (PCCs) depends on filler fraction in a non-linear fashion [55]. This non-linear relationship is mainly caused by the percolation threshold (formation of long-range connectivity in a random system), leading to a sudden increase in thermal conductivity [56,57,58]. However, the influence of the filler on the thermal conductivity increase cannot be accurately predicted based on filler dimensions alone. This is caused by a lacking description of the filler distribution and network formation within the matrix. This lack in understanding hinders the reproducibility and understanding of the thermophysical properties of PCCs.

The addition of thermally conductive fillers can also improve the shape stability of the PCC during thermal cycling [59]. Most studies on filler enhanced organic PCMs focus on describing the change in thermal properties depending on the filler loading and sometimes filler type [17,60,61,62]. The microscopic/mesoscopic boundary condition that fillers should form a thermally conductive network is, however, often not accounted for. We hypothesize that the thermal conductivity of the PCC is not only a function of the thermal conductivity of the filler and matrix, but also a function of the filler distribution and resulting network topology. The dependence of thermal conductivity on network topology is a result of the thermal resistance between matrix–filler and filler–filler surfaces [63,64,65]. More detailed insights on how filler dimensions, aspect ratio and processing affect network structure, thermal properties and shape stability are, however, still lacking.

Here, we aim to fill this knowledge gap by introducing a PCC description which includes the effects of filler networks. To this end, we systematically investigated how interactions between GNP and paraffin, filler loading, GNP size and aspect ratio shape filler networks within the PCM (paraffin) and in turn determine the thermal conductivity of the PCC and its shape stability. This work is intended to develop a solid (colloidal) foundation for the rational design of GNP-enhanced PCMs with tunable structure, stability, and thermal properties.

## 2. Materials and Methods

### 2.1. Materials

Three different graphene nanoplatelet types of varying size and aspect ratio (H25, M25 and M5; see Table 2) were produced by XG Sciences and purchased from Merck Life Science NV (Amsterdam, The Netherlands). Paraffin with a melting temperature of *T*_m_ ≈ 42–44 °C and a density of 0.90 g/cm^3^ was used as PCM (Merck Life Science NV, Amsterdam, The Netherlands). All materials were used without further purification.

### 2.2. Preparation of GNP–Paraffin Composites

**Figure 1:**
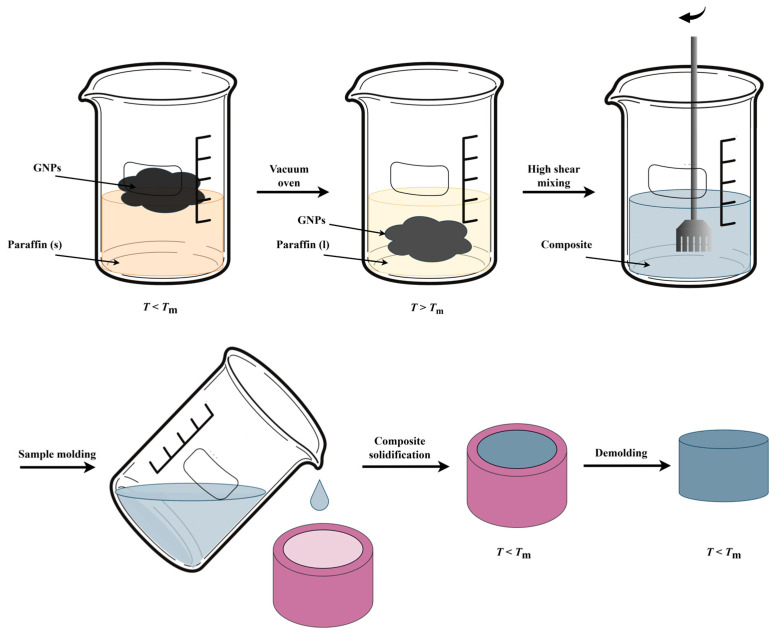
Sketch of the experimental procedure to prepare phase change composites (PCCs) using paraffin (PA) and graphene nanoplatelets (GNPs).

In the first preparation step, a beaker was filled at room temperature (so below *T*_m_ of paraffin) with 40 g paraffin and varying amounts of GNPs to prepare composites with 2, 5, 8 or 10 wt% filler (Figure 1). An upper limit of 10 wt% was chosen, as above this limit, the liquid-to-solid ratio for the H25 GNPs was too low to facilitate proper mixing. Hereafter, the beaker was covered with a thick aluminum foil, which was perforated and subsequently placed in a vacuum oven (Heraeus Vacutherm VT 6025, Hanau, Germany) for 3 h at 70 °C (so *T* > *T*_m_). The resulting paraffin–GNP dispersion was mixed for 5 or 15 min at a shear rate of 4189 s^−1^ (which is far below the exfoliation shear rate of 10,000 s^−1^ to ensure no reduction in GNP sheet size or thickness occurs) [66], using a Ultra Turrax X40/38 (Ystral, Ballrechten-Dottingen, Germany) high shear mixer, equipped with a stator with an internal diameter of 35 mm and a 25 mm rotor at *T* = 70 °C. The liquid composite was then poured into 25 mL plastic cups and cooled to room temperature, resulting in cylindrical PCC samples of 10 mm in height and 15 mm in diameter. Each composite was given a sample code, such as PA-2-M5, indicating a paraffin (PA) composite incorporating 2 wt% of M5 GNP filler. Each composite was prepared and analyzed 3 to 5 times to ensure consistency and reliability of the results.

### 2.3. Paraffin Contact Angle Measurement/Graphene Thin Film Formation

The contact angle of paraffin was measured on four different substrates, glass slides coated with M5 (1), H25 (2), M25 (3) GNPs, and a glass slide (4). To measure the contact angle between paraffin and GNP, GNP thin films were first prepared on glass substrates as described by Arapov et al. [67]. Contact angle measurements were performed using a Dataphysics OCA30 goniometer (Filderstadt, Germany) with OCA20 software and a Dataphysics TPC 100 hot plate, equipped with a UI-3040LE USB 3 camera with 1/3” Sony IMX273 global shutter CMOS sensor (IDS Imaging, Obersulm, Germany). The glass slide containing the thin film was placed on the hotplate set to 65 °C and brought into focus. Simultaneously, the paraffin as well as a glass pipet were heated to 65 °C. The glass pipet was used to collect a droplet of paraffin. While filming and bringing the paraffin droplet (approximately 7 μL) into focus, the glass pipet was used to deposit the droplet onto the GNP thin film. Droplet shape and contact angle were analyzed in individual frames of the video using ImageJ (version 1.54h).

### 2.4. Morphology Characterization

To analyze the distribution of GNPs within the PCM matrix, ideally, the center-to-center distance between GNPs should be measured. However, using stereology it becomes clear that from a 2D cross-section, it is impossible to determine the distances between the centers of each GNP directly. To calculate this distance, one needs another cross-section, for instance, perpendicular to the original one or another assumption, for example, the mean radius of the GNP. As this is a very involved analysis process, we opted instead for approximating the sheet-to-sheet distances from a single random cross-section.

A flat cross-section of composites was created by microtomy using a rotational microtome Leica RM2165 (Leica Biosystems, Buffalo Grove, IL, USA). The samples were cut using a diamond knife at a temperature of −30 °C utilizing a Leica LN21 cryo (Leica Biosystems, Buffalo Grove, IL, USA) unit.

The resulting cross-sections were imaged by scanning electron microscopy (SEM) using a Quanta 3D (Thermo Fisher, Watham, MA, USA) at an acceleration voltage of 4.5–5 kV. Acquired images were quantified in Image J, using an overlaid grid (5 × 5 μm^2^ or 1 × 1 μm^2^) to measure the sheet-to-sheet distance along the grid lines (for details, see Supporting Information Section S1, Figure S1 and Table S1).

To analyze alignment and ordering of GNPs in the composite, an autocorrelation function implemented in DigitalMicrograph (version 3.60.4435) was used. Analysis by autocorrelation is a mathematical tool for finding repeating patterns and orientational asymmetry such as local alignment, i.e., GNP stacking. To distinguish between global and local order, the autocorrelation function was used on representative overview images, and images 4× the lateral size of the GNPs (100 × 100 μm^2^ for M25 and H25, and 20 × 20 μm^2^ for M5) and 2× the lateral size of the GNPs (50 × 50 μm^2^ for M25 and H25, and 10 × 10 μm^2^ for M5).

### 2.5. Thermal Conductivity Measurements

Thermal conductivity *k* was analyzed using the transient plane source method employing a TPS 2500S (HotDisk, Göteborg, Sweden) in combination with a TPS-TP1 temperature platform (Thermtest, Vedigge, Sweden). Before measuring the thermal conductivity of the samples, the calibration of the Kapton 7577 F1 sensor (HotDisk, Göteborg, Sweden) is verified (for details, see Supporting Information Section S2 and Table S2).

The thermal conductivity of the PCC samples was measured at room temperature using the same Kapton 7577 F1 sensor. All samples were measured in duplo for 10 s at 25 mW at least three times, with a wait time of 10 min to equilibrate the sample temperature.

### 2.6. Differential Scanning Calorimetry (DSC) Measurements

The latent heat, supercooling, melting, and crystallization temperatures were determined using differential scanning calorimetry (DSC) using a Q2000 (TA instruments, Newcastle, DE, USA). For all measurements, a heating and cooling rate of 2 °C/min was applied under nitrogen atmosphere [68]. For each sample, three cycles were measured. The data of the first cycle were discarded, as they may contain a thermal fingerprint left by the formation and handling of the composites. The second and third cycles were used to determine the material properties. To determine the crystallization and melting temperature, the onset temperature of the peaks was used [68].

### 2.7. Shape Stability

**Figure 2:**
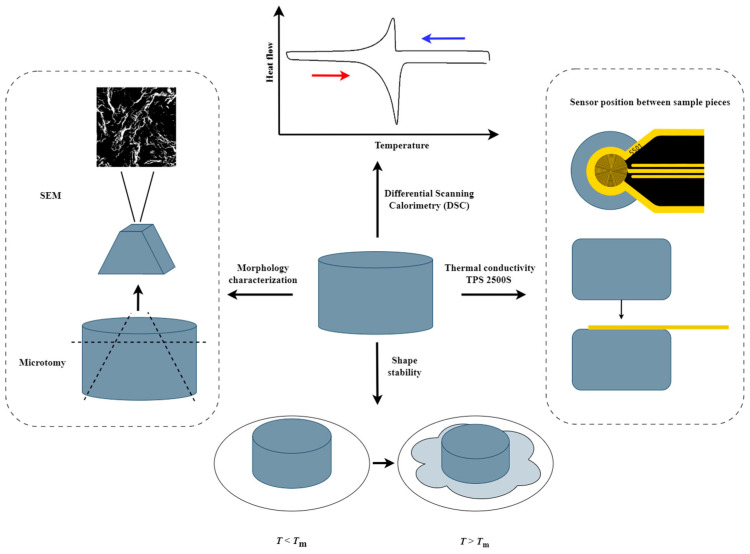
Sketch of the PCC analysis workflow describing the thermal conductivity measurement, shape stability test, morphological characterization and DSC measurements.

We believe shape stability is an important matter to address, as it indicates the potential for phase separation of the PCM from the GNP network, potentially decreasing performance due to changes in sample dimension, shape and GNP network topology. To evaluate shape stability, we followed a standard method in the field [69]. In brief, the method relies on measuring the paraffin loss, i.e., leaking/leakage, from the composite upon melting, indicative of phase separation. Paraffin loss is quantified by measuring the difference in weight of a sample before and after a heating cycle. To this end, the weight of the sample and a filter paper support are first measured. Second, the sample is placed in the center of the filter paper and transferred to a preheated oven (Heraeus Vacutherm VT 6025, Hanau, Germany) at approximately 30 °C above the melting temperature of paraffin in the composite and kept there for 30 min (Figure 2, bottom center). (These conditions are sufficient to completely melt a pure paraffin sample of similar dimensions and previous studies have confirmed that paraffin loss due to evaporation is negligible at these temperatures [70]). Third, after 30 min, the sample is removed from the oven and placed in ambient conditions to cool down and resolidify. Once solidified, the sample is removed from the filter paper, and both the filter paper and sample are weighed again. Any weight loss observed in the sample is accompanied by a corresponding weight gain in the filter paper (further confirming negligible paraffin is lost due to evaporation). During the heating cycle, molten paraffin leaked from the composite and infiltrated the filter paper. The weight change of the composite is expressed as % paraffin loss using the following equation:

% paraffin loss = (1 − (weight sample after melting − weight GNP)/(weight sample before melting − weight GNP)) × 100%

In the above equation, the weight of the GNP is the exact amount of GNP present in the sample, as described in Section 2.2. Here, it is worth noting that no GNP was transferred to the filter paper during the heating cycle.

## 3. Results

The model system used in this study is composed of paraffin and GNPs. In line with the viability criteria mentioned in the introduction, the PCC was made by melt mixing, which is a scalable production method. Three different GNP types of varying lateral size, thickness and aspect ratio were selected for this study (Table 2). It is expected that the aspect ratio and size of the GNPs will have a significant influence on the GNP distribution and, thus, the GNP network topology, as the thermal conductivity has already been shown to be significantly influenced by the GNP type [61,71,72,73]. Each composite in this study is given a sample code PA-X-Y, indicating a paraffin (PA) composite incorporating X wt% of GNP filler of type Y (either M5, H25 or M25). An overview of the analysis workflow of all these composites is provided in Figure 2.

### 3.1. Effects of Mixing Time on GNP Distribution

**Figure 3:**
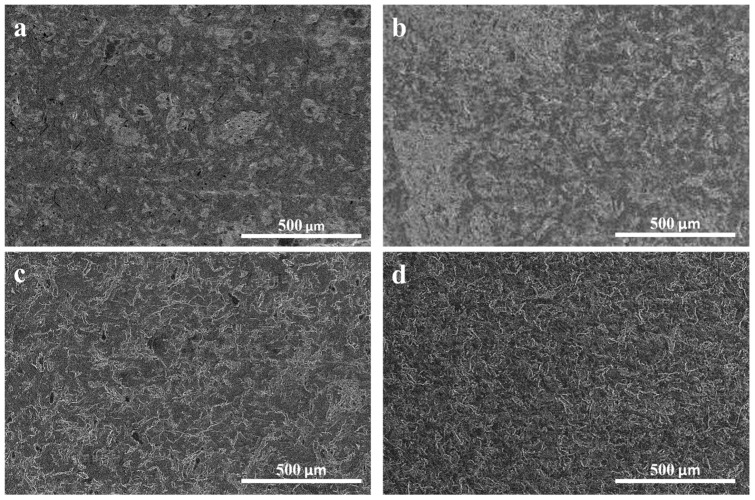
Influence of mixing time on GNP distribution. SEM images of microtomed cross-sections at (**a**,**b**) 5 min mixing time and (**c**,**d**) 15 min mixing for graphene fillers (**a**,**c**) H25 and (**b**,**d**) M25.

To investigate the influence of mixing time on the GNP filler distribution, two composites containing 5 wt% of H25 and M25 GNPs were prepared by high shear mixing for 5 or 15 min. To examine the filler distribution throughout the two composites, cross-sections were prepared by microtomy and imaged by SEM (Figure 3). These SEM images revealed distinct GNP-rich regions at 5 min of mixing, which can be observed as light gray areas (Figure 3a,b). The presence of these GNP-rich areas is most pronounced in PA-5-M25 (Figure 3b), indicating that M25 sheets are harder to disperse at shorter mixing times. However, by increasing the mixing time to 15 min, no graphene-rich areas were observed anymore for either of the PA-5-H25 and PA-5-M25 composites (Figure 3c,d). This observation suggests that a mixing time of 15 min is sufficient to homogeneously disperse these two types of GNPs in the paraffin matrix.

Interestingly, the filler type does not influence the mixing time required to achieve a well-dispersed filler network. Another clear indication of a well-dispersed filler network being formed after a certain mixing time is that the thermal conductivity of the composite reaches a plateau (for details, see Supporting Information Section S3 and Figure S2). In this context, it is important to note that the filler type determines the maximum achievable thermal conductivity, as will be discussed in more detail in Section 3.3.

### 3.2. Melting and Crystallization Temperatures and Latent Heat

**Figure 4:**
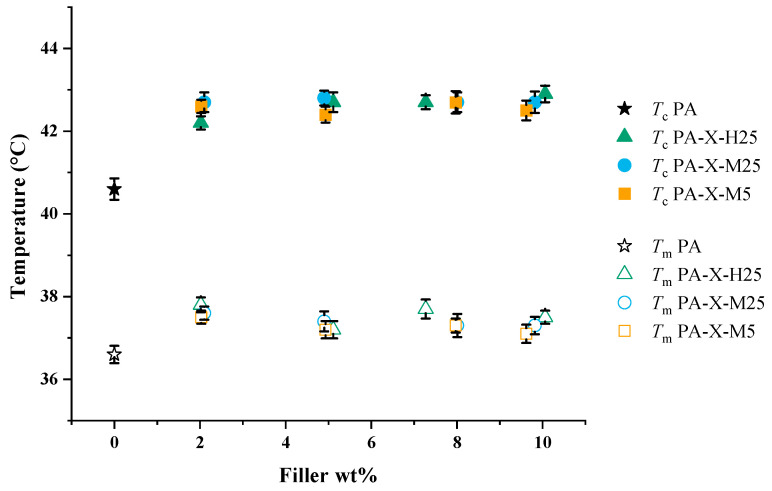
Melting (open symbols) and crystallization (filled symbols) temperatures of pure paraffin and PCCs at varying filler fractions (wt%). Furthermore, *T*_c_ refers to the temperature at the onset of crystallization and *T*_m_ to the onset temperature of melting.

Next, we studied by DSC how the addition of GNPs to paraffin mediates the melting temperature, crystallization temperature and latent heat. In Figure 4, we present the measured temperatures for melting (open symbols) and crystallization (filled symbols) that correspond to the onset of melting and crystallization, respectively. It is apparent that both the melting and the crystallization temperatures for pure paraffin are lower than for composites (Figure 4), indicating that the GNPs probably act as a surface for heterogeneous nucleation of paraffin. Notably, the filler fraction and type of filler do not have a significant effect on the melting and crystallization temperature of the composite. The fact that GNPs act as a nucleating surface for paraffin is not unexpected, since the GNPs cause irregularities in the PCM and provide surfaces for heterogeneous nucleation.

**Figure 5:**
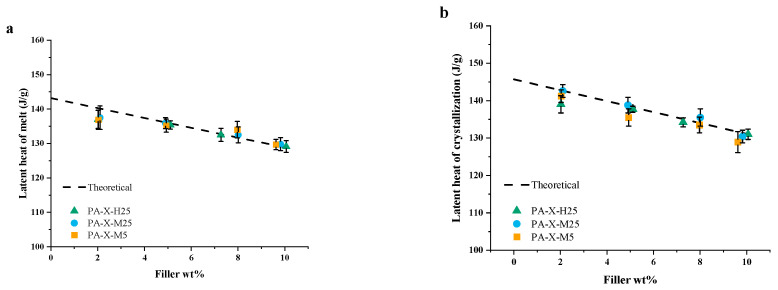
DSC data from pure paraffin and the PCCs: (**a**) latent heat of melting and (**b**) latent heat of crystallization.

Upon the addition of GNP and with increasing filler fraction, it is apparent that there is a clear, nearly linear decrease in the latent heat of melting (Figure 5a) as well as in the latent heat of fusion (Figure 5b), both being independent of the GNP type. This linear decrease in the latent heat of melting, as well as the latent heat of fusion, as a function of the filler mass fraction of the PCM, $\varphi_{m}$, is expected, as it agrees with the theoretical latent heat of these composites:

Δ*H_c_* = *φ_m_* · Δ*H_m_* (1)

Here, Δ*H*_c_ and Δ*H*_m_ are the latent heat of the composite and PCM, respectively.

### 3.3. Effect of Filler Aspect Ratio and Amount on Thermal Conductivity

**Figure 6:**
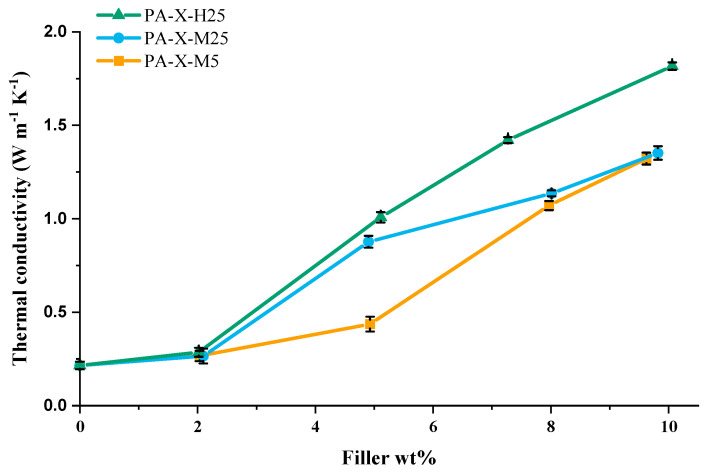
Thermal conductivity of PCCs for various GNP filler types (H25, M25 and M5; see Table 2 for details) as a function of GNP filler weight fraction.

In Figure 6, a detailed overview of the influence of filler type (aspect ratio and size) and filler fraction on the measured thermal conductivity is shown. From these results, it can be inferred that there are three different regimes (≤2 wt%, 2–5 wt%, ≥5 wt%) that need to be considered. The first regime, ≤ 2 wt%, shows no effect of aspect ratio or size on the thermal conductivity.

In the second regime, 2–5 wt%, the lateral size of the GNPs exhibits a prominent influence on the thermal conductivity of the composites. Specifically, composites incorporating 5 wt% GNPs with a lateral size of 25 µm show a significantly higher thermal conductivity compared to those containing GNPs with a lateral size of 5 µm, with values of 1.01 W·m^−1^·K^−1^ and 0.44 W·m^−1^·K^−1^, respectively. The observed influence of lateral size on the thermal conductivity can be understood in terms of a decreased number of sheet-to-sheet contacts for thermal transport across a specific distance, similar to what has been observed for electrical conductivity in printed graphene conductors [74]. As a result, composites containing 25 µm GNP fillers exhibit a more rapid initial increase in thermal conductivity due to the decreased number of sheet-to-sheet contacts than composites containing 5 µm GNP fillers.

At higher GNP concentrations (regime 3, >5wt%), the GNP thickness seems to become the dominant factor determining the thermal conductivity. Notably, an increase in the number of graphene layers in the GNP leads to an increased thermal conductivity, as observed in the composites with 8 and 10 wt% GNP filler. A similar trend can be seen in the data published by Warzoha et al. [71]. Figure 6 clearly shows that the H25 filler leads to the highest thermal conductivity at all filler fractions studied.

This result seems counterintuitive from a percolation threshold perspective because the M25 fillers are half as thick (7 nm) as the H25 fillers (15 nm), and consequently, at a given filler fraction, the composite will contain twice as many M25 sheets compared to H25 sheets. This would mean that in the M25 composite, GNP fillers would form a percolating network at a lower weight fraction and, in principle, should outperform the H25 composite. This indicates that highly performing filler networks not only depend on the number of sheets in the composite but other factors too. Therefore, to explain this thermal conductive behavior, we need to look at the interactions between the matrix and the filler, the detailed distribution of the filler throughout the matrix (network topology), and filler-filler contacts.

### 3.4. Paraffin-GNP Interactions

The interaction between the GNP and paraffin plays an important role in the filler distribution as well as the resulting composite properties. When filler and matrix are chemically compatible, good wetting results in a lowering of the thermal resistance across the GNP-paraffin interface and will result in a lower chance of phase separation due to chemical incompatibility. Furthermore, it was suggested in the literature that the strong attractive forces between graphene/CNT and paraffin cause an increase in latent heat [75,76]; however, this trend is not observed for our composites (Figure 5a,b).

**Figure 7:**
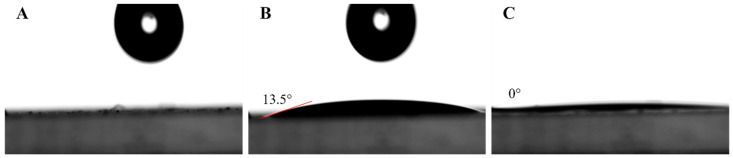
Contact angle measurement of molten paraffin droplet on GNP thin film. (**A**) GNP thin film before droplet placement; (**B**) 0.1 s after droplet placement; and (**C**) 3 s after droplet placement, showing transient wetting.

To investigate the wettability of the GNP filler by paraffin, the contact angle was measured at 65 °C (Figure 7), which is above the melting temperature of paraffin. To determine the contact angle of paraffin with GNPs, a thin film of M25 GNPs was formed by self-assembly of a graphene thin film at an oil/water interface [67]. After the molten paraffin droplet was placed on the surface, the droplet spread out quickly to a contact angle of 13.5°, indicating good wetting behavior (Figure 7B). Within three seconds, the molten paraffin spread out even more, resulting in a contact angle of 0°, i.e., complete wetting on the surface (Figure 7C). This behavior is also observed for thin films formed from M5 and H25 GNPs (for details, see Supporting Information Section S4 and Figure S3). This wetting behavior clearly indicates a strong affinity between paraffin and graphene. This also means that GNPs are expected to be completely wetted by paraffin during the formation of the PCCs. Paraffin layers surrounding graphene sheets in the molten state have also been observed in simulations [75].

The strong attractive interactions between paraffin and graphene have been previously shown to have a positive influence on the shape stability of the composite and, thus, the amount of paraffin leakage [59]. We found a similar trend, as shown in Table 3. There is a clear decrease in the paraffin loss and, thus, an increase in the shape stability upon increasing the filler content. Importantly, shape stability also increases with increasing GNP size and aspect ratio. If the strong attractive interaction between paraffin and graphene is the only factor that influences shape stability, it would be expected that increasing the available surface area of the GNP would result in a decrease in paraffin leakage. This is, however, not what we observe for M5 and M25 fillers, which have a similar specific surface area (Table 2) but show significantly different shape stability. Therefore, the interaction between graphene and paraffin cannot be the only contributing factor to the shape stability. We hypothesize that the filler distribution throughout the composite also has a significant effect on shape stability. To gain more insight into the filler distribution, we will first determine the GNP percolation threshold in paraffin (Section 3.5) and then investigate the sheet-to-sheet distance and overlap distance of the sheets both theoretically and experimentally (Section 3.6).

### 3.5. Percolation Threshold

Assuming we have perfect randomly mixed composites, we can theoretically estimate the percolation threshold for platelets of different dimensions. This is performed by treating the GNPs in first approximation as *N* hard colloidal platelets, with diameter *D* [m] and thickness *L* [m], in a total volume V [m^3^]. The volume fraction of hard platelets equals *ϕ* = π*NLD*^2^/4*V*. The GNPs studied have a high aspect ratio *D/L*, as well as a high surface area, resulting in a large, excluded volume, which drives the transition from the isotropic (completely random particle distribution) towards the nematic state (introduction of one-dimensional order) already at low particle concentrations. This isotropic–nematic phase transition occurs at filler fractions close to the percolation threshold [77]. A theoretical prediction of the isotropic (I)-to-nematic (N) phase transition of hard colloidal platelets has, for instance, been proposed by Wensink and Lekkerkerker [78]. They found that the onset of this phase transition (hence, close to percolation) occurs when the dimensionless particle concentration c = *ND*^3^/*V*, with *N*, the number of hard platelets in a volume (*V*), exceeding a value close to 4, c = (4/π)*φD*/*L*. Some values for the dimensionless particle concentrations c are given in Table S3 for the three different GNPs for various filler fractions. Obviously, already at 2 wt% of GNP, the theoretical onset value of percolation, which was 4, was surpassed for all composites. 

The volume fraction at the isotropic–nematic phase transition (*φ*_IN_) can be estimated from:

*φ*_IN_ = π · *L/D* (2)

In Table 4, the calculated volume fraction for an I-N phase transition using Equation (2) is shown in the first column for the three discussed composites. The calculated values show a significant deviation from our experimentally obtained values (Table 4, column 2) that places the transition for all three composites at 2 wt% filler fraction, i.e., 0.009 volume fraction. The observed difference likely originates from variations in filler aspect ratio, i.e., deviations in GNP thickness from nominal thickness. To this end, Equation (2) can also be utilized to calculate the GNP thickness (Table 4, column 3) based on our experimental I-N phase transition volume fraction. This calculated GNP thickness is 4.8 to 14.4 times higher than the manufacturer-provided nominal value. For comparison, we added the experimental data by Warzoha et al. [71] to Table 4 column 4 and also calculated the corresponding GNP thickness, which is shown in Table 4, column 4. Also, these data show a similar deviation to our data between the experimental and theoretical volume fraction at the isotropic–nematic phase transition. To resolve the matter, a local analysis of the GNP thickness in PA-5-M25 was carried out using cryo FIB-SEM cross-sections, which show that the actual GNP thickness is close to the calculated GNP thickness based on an I-N phase transition at 2 wt% (for details, see Supporting Information Section S6, Figure S4). An increased sheet thickness also affects the percolation threshold, since the increased sheet thickness raises the percolation threshold by shifting the isotropic-nematic phase transition to higher volume fractions, as the platelet thickness is directly proportional to this transition (Equation (2)). A table on the dimensionless particle using experimental values is provided in Supporting Information Section S5, Table S4. 

The similarity in theoretical sheet thickness for H25 and M25 also raises some questions regarding the observed difference in thermal conductivity for PA-X-M25 and PA-X-H25 (Figure 6). To understand where this difference originates from, one needs to investigate the distribution of GNPs throughout the composite.

### 3.6. Sheet-to-Sheet Distance, Mean Overlap Distance and Network Topology

**Figure 8:**
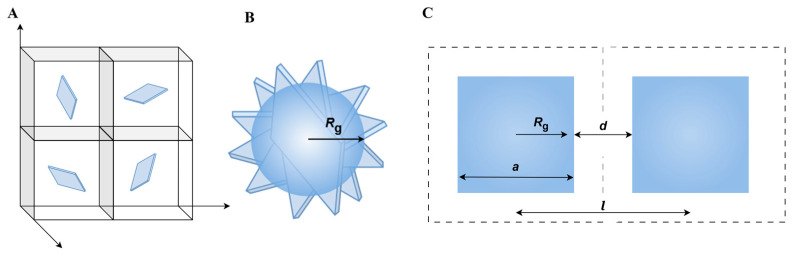
Schematic drawing of (**A**) the ideal 3D distribution of GNPs in identical volume elements; (**B**) randomly oriented square and its radius of gyration; and (**C**) definition of average overlap distance and average sheet-to-sheet distance.

To quantify the distribution of the GNPs throughout the matrix, we estimate the average sheet-to-sheet distance l [µm] as well as the average overlap distance d [µm] (Figure 8 and Table 5). In the following pages, we first define these terms and explain how we estimate the averages of the sheet-to-sheet and the overlap distances. Hereafter, the results will be presented and discussed.

The sheet-to-sheet distance can be described as the distance between the mean centers of mass of neighboring GNPs. This description assumes a homogeneous GNP distribution throughout the matrix, as obtained after 15 min of high shear mixing. Consequently, the composite is treated in first approximation as being built of cubic volume elements, wherein each element’s center is occupied by one filler particle (Figure 8A). This means that the total number of volume elements present corresponds to the number of filler particles, i.e., number of GNPs in the composite. 

We assume that the GNPs are in first approximation flat squares with edge length *a* = 5 µm (M5) or 25 µm (M25, H25) with a high aspect ratio, covering an area of 25 µm^2^ (M5) or 625 µm^2^ (M25, H25). Furthermore, we assume that the GNPs are randomly dispersed, allowing them to adopt any orientation (Figure 8B), so they can occupy a volume that is characterized by the radius of gyration *R*_g_ [m]. For a plate-based system, the radius of gyration is related to the edge length a through:

*R*_g_ = 1/2^−1/3^ · *a* (3)

As a result, the length of a single cubic element and thus the average sheet-to-sheet distance *l* [m] (Figure 8C) is related to *R*_g_ and the average overlap distance *d* [m] (Figure 8C) as:

*l* = 2 *R*_g_
*+ d* = (*V_cell_*)^1/3^ (4)

The volume of the cubic volume element *V_cell_* [m^3^] (Figure 8A) can be calculated from:

*V*_*cell*_ = *V/N* (5)

where *N* is the number of GNP sheets in the composite and *V* [m^3^] is the total volume of the composite. 

These calculations reveal, as expected, a decrease in sheet-to-sheet distance as the filler wt% increases (Table 5). Furthermore, the sheet-to-sheet distance of the M5 filler is significantly smaller than that of the M25 filler. This difference arises from the fact that M5 sheets are smaller, having an edge length that is five times smaller than that of the M25 sheets, 5 µm and 25 µm, respectively (Table 2). This size difference means that there are 25 times more sheets present in the M5 composites compared to M25 at a given filler wt%, thereby increasing the number of identical volume elements and, thus, reducing the sheet-to-sheet distance. 

**Figure 9:**
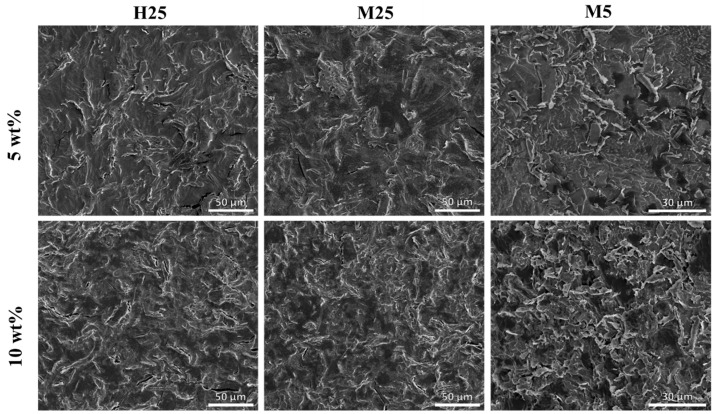
SEM images of microtomed cross-sections of composites showing distribution of GNP fillers (bright lines) in paraffin matrix (gray).

Having established a theoretical estimate for the average sheet-to-sheet distance, experimental validation is required. While the average sheet-to-sheet distance, as indicated in Figure 8C, cannot be directly measured, it can be approximated using a random cross-section of the composite. To this end, cross-sections of the composite were prepared by microtomy, imaged in SEM, and analyzed quantitatively. (Further details are provided in the experimental section and Supporting Information Section S1). A first visual impression on the distribution of GNPs in different composites is shown in Figure 9. As predicted from the theoretical model, PA-5-M5 and PA-10-M5 show a significantly higher number of sheets compared to the composites containing H25 or M25 GNPs. Clear differences between PA-5-H25, PA-5-M25 and PA-10-H25, PA-10-M25 are not visible, which is expected since the theoretical sheet-to-sheet distances of these composites are comparable (Table 5). When measuring the sheet-to-sheet distance by SEM (for details, see Supporting Information Section S1), in general, larger values than the theoretical estimates are found. This difference is explained by, e.g., M25 sheets having an average sheet thickness of 83 ± 7.4 nm as opposed to the 7 nm, as suggested by the manufacturer (Table 2). When calculating the sheet-to-sheet distance of PA-X-M25 with this observed sheet thickness, the sheet-to-sheet distance is increased by a factor of 2.28, resulting in a sheet-to-sheet distance of 14.26 µm and 11.19 µm for PA-5-M25 and PA-10-M25, respectively, which is in line with the experimental sheet-to-sheet distance for these composites.

To form a stable network and therefore create shape stability, the GNP fillers need to be in contact with each other. This means that there needs to be an average overlap of the GNPs, which can be expressed as the overlap distance d. For overlap to occur, the average overlap distance d should be negative, as can be deduced from Figure 8 and Equation (4). When looking at the experimental overlap distance (Table 5), we see two composites with an overlap distance that does not allow the GNPs to touch each other, namely PA-5-M5 and PA-5-H25. Interestingly, these two composites show paraffin leakage upon melting and are therefore not shape-stable (Table 3). This correlation between shape stability and overlap distance indicates that we can predict the shape stability of the composite based on our model of the filler distribution in the composite.

### 3.7. What Governs Thermal Conductivity?

While overlap distance seems an excellent measure to explain network formation and shape stability of composites, it cannot explain the difference in thermal conductivity between PA-X-M25 and PA-X-H25, as presented in Figure 6. In addition, one needs to consider effects such as orientational alignment of GNPs when being above the weight fraction for the isotropic (I)-to-nematic (N) phase transition mentioned above. To this end, we utilized SEM image analysis by autocorrelation, which is a mathematical tool to investigate repeating patterns or directional anisotropy in images. 

**Figure 10:**
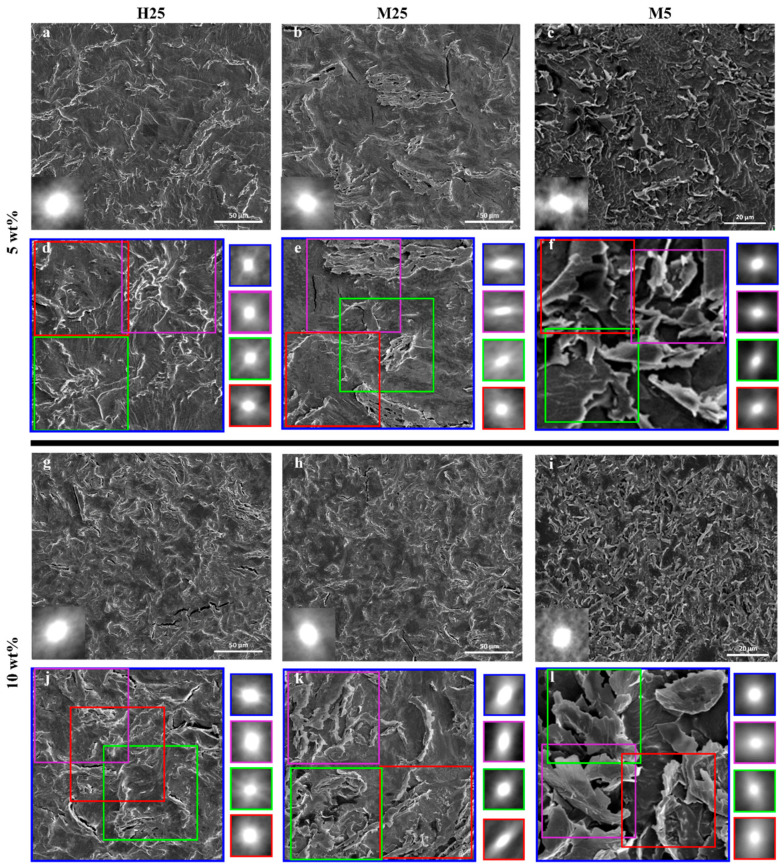
SEM images of composites with autocorrelation shown in insets to analyze orientational order: (**a**,**d**,**g**,**j**) PA-X-H25; (**b**,**e**,**h**,**k**) PA-X-M25 and (**c**,**f**,**i**,**l**) PA-X-M5. (**a**–**c**,**g**–**i**) A 100 × 100 μm area within (**d**,**j**) PA-X-H25; (**e**,**k**) PA-X-M25 and a 20 × 20 μm area within (**f**,**l**) PA-X-M5 composite. The smaller colored squares in the SEM image are a 50 × 50 μm area (for PA-X-H25 and PA-X-M25) and 10 × 10 μm area (for PA-X-M5). The regions have been chosen to omit the effect of the surface cracks on the autocorrelation analysis. The four squares on the side of the image show the central autocorrelation spot for each of the corresponding SEM images.

Overview SEM images of all composites with 5 wt% (Figure 10a–c) or 10 wt% (Figure 10g–i) reveal a central autocorrelation spot (insets) that is close to spherical, indicating directional isotropy over large areas. However, the presence of ordered domains at smaller length scales also needs investigating to better understand the effects of alignment/stacking of GNPs on thermal conductivity.

Upon analyzing areas corresponding to 4 × 4 or 2 × 2 by autocorrelation, the GNPs’ lateral size reveals clear differences. For PA-5-H25 (Figure 10d), PA-10-H25 (Figure 10j), PA-5-M5 (Figure 10f), and PA-10-M5 (Figure 10l), a close-to-spherical central spot is observed, indicating that no alignment/stacking at any length scale occurs. In contrast, a clear elongation of the central autocorrelation spot is noted for PA-5-M25 (Figure 10e) and PA-10-M25 (Figure 10k) at smaller length scales. This indicates that there is orientational alignment or stacking of GNPs within the composite. We hypothesize that this stacking of M25 GNPs is the reason for limited gains in thermal conductivity when increasing from 5 to 10 wt% (Figure 6).

Based on the above findings, we infer that three regions exist in the thermal conductivity vs. filler fraction curve (Figure 6). In the first region (0 to 2 wt% for PA-X-H25, PA-X-M25 and 0 to 5 wt% for PA-X-M5), there is a minimal increase in thermal conductivity, as the percolation threshold has not yet been reached. The second region shows a significant increase in thermal conductivity upon increasing filler wt%. This can be observed for PA-X-H25 from 2 to 10 wt%, for PA-X-M25 from 2 to 5 wt%, and from 5 to 10 wt% for PA-X-M5. An important aspect for this significant increase in thermal conductivity is the directional isotropy on all length scales at these filler wt%, as shown with the autocorrelation function (Figure 10). The third region is characterized by GNP stacking at smaller length scales, which is visualized using autocorrelation (Figure 10e,k) and results in a minimal increase in thermal conductivity by increasing the filler wt%, which is observed for PA-X-M25 from 5 to 10 wt%. These three regions strongly suggest that thermal conductivity is not solely determined by the filler fraction, but importantly also governed by the distribution and orientation of the filler particles.

## 4. Conclusions and Outlook

In this manuscript, we investigated the effect of GNP filler size, aspect ratio and filler fraction on the resulting thermal conductivity, shape stability and network topology of paraffin-based PCMs. As expected, the thermal conductivity increases with increasing filler loading, and optimal performance depends on sufficiently long mixing times that create macroscopically homogeneous composites. 

Zooming into the mesoscopic and nanoscopic distribution of GNP fillers provides a colloidal perspective on the network topology and its relation to thermal performance and shape stability. To this end, we utilized theoretical estimates and SEM-based validation of the GNP filler distribution throughout the matrix at filler loadings from 2 to 10 wt%. Clearly, with increasing filler loading, the GNP sheet-to-sheet distance decreases and sheet overlap occurs, which results in the formation of a shape-stable composite. However, it is not the absolute sheet-to-sheet distance that determines the shape stability. Instead, the distance minus twice the radius of gyration of the filler provides a guide, as overlap between platelets is needed to form a shape-stable network.

Concentrating on thermal conductivity, we observe that it is not the network with the highest GNP aspect ratio that has the highest thermal conductivity, but rather the network with an optimized aspect ratio for a given volume fraction. Furthermore, we suggest that by analyzing the filler network topology, we can explain the thermal conductivity behavior of the PCCs based on three characteristic regions. These regions can be defined as (1) pre-filler percolation, (2) random network formation and (3) GNP stacking.

These findings highlight the importance of establishing a colloidal perspective of PCM composites for designing the next generation of shape-stable and highly thermally conductive PCMs based on optimized volume fraction, filler size and aspect ratio. We believe the principles presented here can provide insight into any PCM matrix and 2D filler system. However, the effect of material properties on the thermal conductivity and shape stability, such as polarity, viscosity and wettability, are not yet well understood. Therefore, similar research using fatty acids, fatty alcohols and other 2D fillers is needed.

## Figures and Tables

**Table 1:** Advantages and disadvantages of inorganic, binary and organic heat storage materials [4].

	Inorganic Material	Binary Material	Organic Material
**Examples**	Salt hydrates (Na_2_SO_4_ 10H_2_O, C_2_H_3_NaO_2_) Metals (Gallium, Bi_49_In_21_Pb_18_Sn_12_)	Inorganic–inorganic (NH_2_CONH_2_ +NH_4_NO_3_) Inorganic–organic (Mg(NO_3_)_2_ •6H_2_O + glutaric acid) Organic–organic (Palmitic acid–Stearic acid)	Paraffin Fatty acids Alcohol Ester Polyethylene glycol
**Advantages**	Non-flammable High heat of fusion (100–200 kJ/kg) Good thermal conductivity (0.5–1.5 W/mK)	Wide range of phase change temperatures Chemically and thermally stable High heat of fusion (100–230 kJ/kg) Little supercooling	Non-corrosive Chemically and thermally stable Little supercooling High heat of fusion (130–260 kJ/kg) Non-toxic
**Disadvantages**	Corrosive High supercooling Metastability (hysteresis) Thermal instability High density	Low thermal conductivity Expensive Limited data available on thermophysical properties	Low thermal conductivity Flammable Relatively large volume change (up to 15 %)

**Table 2:** GNP aspect ratio, size and thermal conductivity as provided by the manufacturer.

Graphene Source	Lateral Size/Diameter (μm)	Thickness (nm)	Surface Area (m^2^/g)	Aspect Ratio	Thermal Conductivity (W·m^−1^·K^−1^)
**M5**	5	7	150	625	3000
**H25**	25	15	80	1667	3000
**M25**	25	7	150	3125	3000

**Table 3:** Results of shape stability test listing % paraffin weight loss from composite due to leakage after heating to 30 °C above meting temperature for 30 min.

Wt%	M5	H25	M25
2	89	38	18
5	43	12	0
8	12	0	0
10	0	0	0

**Table 4:** Theoretical and experimental GNP volume fraction for I-N phase transition as well as the calculated GNP thickness derived from Equation (2).

Filler	Theoretical $\varphi_{\mathrm{IN}}$	Experimental $\varphi_{\mathrm{IN}}$ (2 wt%)	Calculated GNP Thickness (nm)	^a^ Experimental $\varphi_{\mathrm{IN}}$	^a^ Calculated GNP Thickness (nm)
H25	0.0018	0.009	72	0.025	199
M25	0.00088	0.009	72	0.009	72
M5	0.0044	0.009	14	0.009	72

^a^ see Warzoha et al. [71].

**Table 5:** Theoretical and measured values for average sheet-to-sheet distance and experimental overlap distance.

Filler wt%	Theoretical Sheet-to-Sheet Distance (µm)	Measured Sheet-to-Sheet Distance (µm) ± Stdev (µm)	Experimental Overlap Distance (µm)
PA-2-H25	10.901		
PA-5-H25	7.954	15.70 ± 14.69	1.27
PA-8-H25	7.035		
PA-10-H25	6.274	12.48 ± 8.61	−1.95
PA-2-M25	8.354		
PA-5-M25	6.256	14.11 ± 12.95	−0.32
PA-8-M25	5.274		
PA-10-M25	4.909	11.48 ± 9.25	−2.95
PA-2-M5	2.888		
PA-5-M5	2.136	2.91 ± 0.98	0.02
PA-8-M5	1.807		
PA-10-M5	1.691	1.66 ± 0.71	−1.23

## Data Availability

The original contributions presented in this study are included in this article. Further inquiries can be directed to the corresponding author.

## Note

1 For clarification we consider eutectic mixtures as binary eutectic mixtures, in contrast to previous work where the term eutectic was used also in cases where no eutectic point was reached, and/or literature shows no consensus on the location of the eutectic point [17,18,19].

## Supplementary Materials

The following supporting information can be downloaded at: https://www.mdpi.com/article/10.3390/nano15080587/s1, Figure S1: Example of sheet-to-sheet distance measurement using (a) an SEM image of PA-5-H25 and a (b) SEM image of PA-5-H25 with a 1 × 1 μm grid overlay; Table S1: Number of measurements for the estimation of the sheet-to-sheet distance; Table S2: Thermal conductivity (*k*) and standard deviation (σ) data of reference material to verifying the calibration of the used sensor; Figure S2: Influence of mixing time on thermal conductivity. Thermal conductivity data of PA-5-H25 and PA-5-M25 at 5, 15 and 30 min mixing time and a pure paraffin refence; Figure S3: Contact angle measurement of molten paraffin droplet on GNP thin films and glass after 0 s, 0.1 s, and 3 s; Table S3: Theoretical dimensionless particle concentration *c* for composites at different wt% and filler aspect ratio, for the manufacturer provided GNP geometry; Table S4: Corrected dimensionless particle concentration *c* for composites at different wt% and filler aspect ratio, with a corrected GNP sheet thickness; Figure S4: SEM image of cryo FIB cross section of PA-5-M25.

## Abbreviations

The following abbreviations are used in this manuscript:

CNT	Carbon Nanotube
DSC	Differential Scanning Calorimetry
EG	Expanded Graphite
GNP	Graphene Nano Platelet
H25	Graphene nanoplatelet type, 25 µm wide and 15 nm thick
LHS	Latent Heat Storage
M25	Graphene nanoplatelet type, 25 µm wide and 7 nm thick
M5	Graphene nanoplatelet type, 5 µm wide and 7 nm thick
PA	Paraffin
PCC	Phase Change Composite
PCM	Phase Change Material
SEM	Scanning Electron Microscope
SHS	Sensible Heat Storage
TES	Thermal Energy Storage
THS	Thermal Heat Storage
