# Supplementary material for: Graphene Nanoplatelet Distribution Governs Thermal Conductivity and Stability of Paraffin-Based PCMs
Source: Nanomaterials (Basel). 2025 Apr 11;15(8):587. doi: 10.3390/nano15080587 (PMC12029283; doi:10.3390/nano15080587)
Supplement: Supplementary file 1 [file nanomaterials-15-00587-s001.zip › nanomaterials-3537943-supplementary.pdf]

Supporting Information

Graphene Nanoplatelet Distribution Governs Thermal Conductivity and Stability of Paraffin-Based PCMs

Levina E. A. Wijkhuijs [1,2,3], Pauline Schmit [1,4], Ingeborg Schreur-Piet [1,4], Henk Huinink [3,5], Remco Tuinier [1,2] and Heiner Friedrich [1,2,3,4,*]

[1] Laboratory of Physical Chemistry, Department of Chemical Engineering and Chemistry, Eindhoven University of Technology, P.O. Box 513, 5600 MB Eindhoven, The Netherlands

[2] Institute for Complex Molecular Systems, Department of Chemical Engineering and Chemistry, Eindhoven University of Technology, P.O. Box 513, 5600 MB Eindhoven, The Netherlands

[3] Eindhoven Institute for Renewable Energy Systems (EIRES), Eindhoven University of Technology, University of Technology, P.O. Box 513, 5600 MB Eindhoven, The Netherlands

[4] Center for Multiscale Electron Microscopy (CMEM), Department of Chemical Engineering and Chemistry, Eindhoven University of Technology, University of Technology, P.O. Box 513, 5600 MB Eindhoven, The Netherlands

[5] Transport in Permeable Media, Department of Applied Physics and Education, Eindhoven University of Technology, University of Technology, P.O. Box 513, 5600 MB Eindhoven, The Netherlands

* Correspondence: h.friedrich@tue.nl

Section S1 Sheet-to-sheet distance measurement example

The sheet-to-sheet distance measurements were executed by placing a 5x5 µm or 1x1 µm grid on top of the SEM image. In the example in Figure S1a a SEM image of PA-5-H25 is shown. Firstly, the length of the scalebar is measured, as can be seen by the yellow line. The length of this line is given in the number of pixels it contains (both horizontal and vertical). Secondly, on top of the SEM image a 1x1 µm grid is placed indicated by the light blue lines (Figure S1b). Hereafter, the sheet-to-sheet distance is measured along the grid-lines. An example (both vertical and horizontal) of this is visualized by the red lines. The length of these lines is also provided in the number of pixels they contain. Finally, the number of pixels is converted into a length given in µm. By this method we calculated the average of the distances between the GNPs measured over the gridlines (as visualised in Figure S1b)

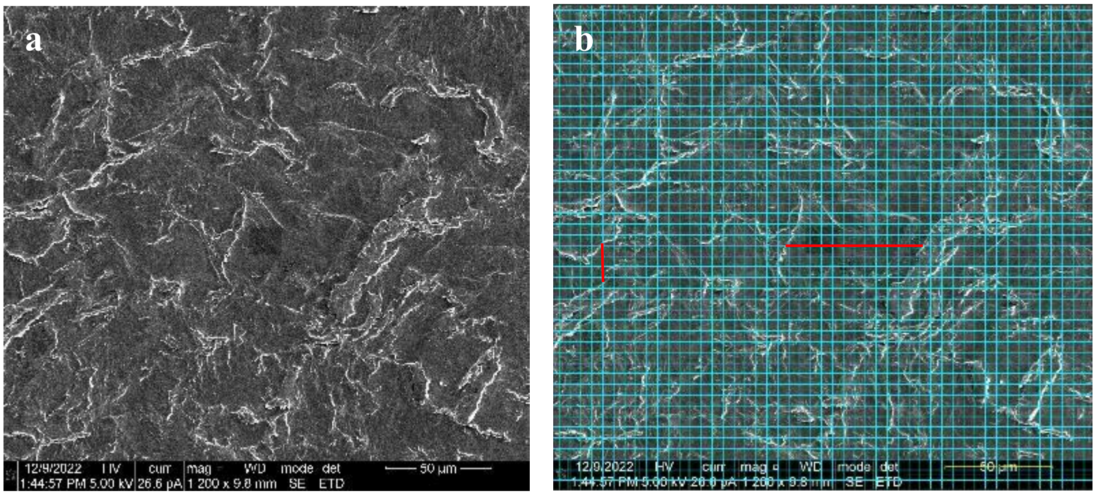

Figure S1. Example of sheet-to-sheet distance measurement using (a) an SEM image of PA-5-H25 and a (b) SEM image of PA-5-H25 with a 1x1 µm grid overlay.

Tabel S1: Number of measurements for the estimation of the sheet-to-sheet distance.

Sample	Number of measurements (n)
PA-5-H25	852
PA-10-H25	791
PA-5-M25	628
PA-10-M25	747
PA-5-M5	473
PA-10-M5	582

Section S2 Calibration verification TPS 2500S sensor

The calibration verification was done by measuring the thermal conductivity of stainless steel and polystyrene reference sample provided by the manufacturer. These two test samples cover both a higher and lower thermal conductivity, as compared to our samples. The average thermal conductivity was calculated as an average of 10 measurements. All measurements (Table S1) of the manufacturer provided test samples were within 1% of the data provided by the supplier, ensuring accuracy of the measurement method.

Table S2. Thermal conductivity (*k*) and standard deviation (σ) data of reference material to verifying the calibration of the used sensor.

	**Manufacturer data**		**Measurement data**			
	5501		5501 F2		7577 F1	
	k ($W{\cdot}m^{-1}{\cdot}K^{-1}$)	σ (%)	k_{av} ($W{\cdot}m^{-1}{\cdot}K^{-1}$)	σ (%)	k_{av} ($W{\cdot}m^{-1}{\cdot}K^{-1}$)	σ (%)
**Stainless steel**	14.13	0.12	14.14	0.025	14.18	0.042
**Polystyrene**	0.031	1.8	0.032	0.005	0.032	0.003

Section S3 Effect of mixing time on thermal conductivity

The observed difference in filler distribution within the composite, caused by a variation in mixing time, resulted in notable differences in thermal conductivity, i.e., an increase from 0.67 $W \cdot m^{-1} \cdot K^{-1}$ to 0.88 $W \cdot m^{-1} \cdot K^{-1}$ for PA-5-M25(Figure S1). Mixing for longer times did not further increase the thermal conductivity of the composites, indicative that a steady state was reached, the variations were within the measurement error. The thermal conductivity not only changes by increasing the mixing time, but also by changing the filler aspect ratio.

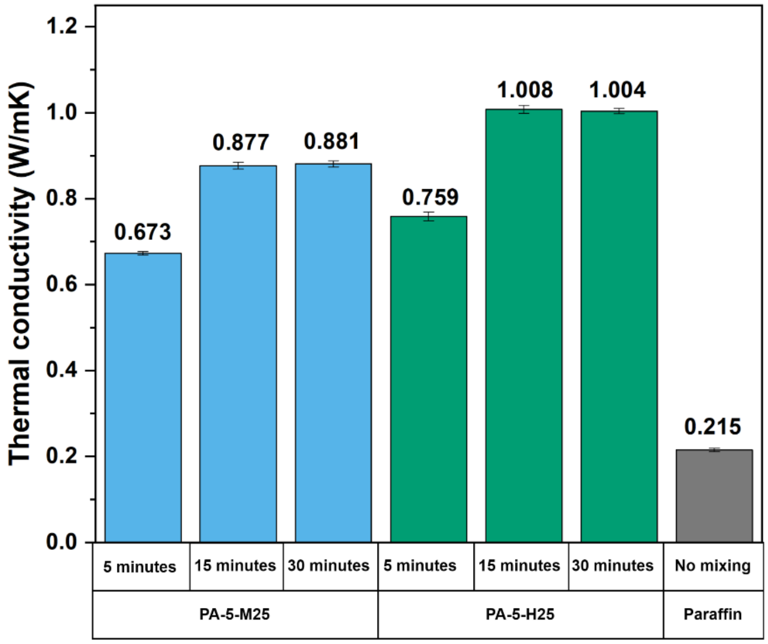

Figure S2. Influence of mixing time on thermal conductivity. Thermal conductivity data of PA-5-H25 and PA-5-M25 at 5, 15 and 30 min mixing time and a pure paraffin refence.

Even when using fillers with similar lateral sizes, a notable difference in thermal conductivity is seen between PA-5-M25 to PA-5-H25 (Figure S2) with thermal conductivity increasing from 0.877 $W \cdot m^{-1} \cdot K^{-1}$to 1.008 $W \cdot m^{-1} \cdot K^{-1}$, respectively.

Section S4 Contact angle measurement

Contact angle measurements were performed on four different substrates. The paraffin droplet behaved similarly on all three different GNP substrates, showing a droplet with a contact angle of approximately 13.5° after 0.1 second and complete wetting after 3 seconds. However, when the same measurement was performed on a glass slide, the droplet shape and size did not change between 0.1 and 3 seconds. Indicating a clearly different behavior between the glass and the GNP substrates.

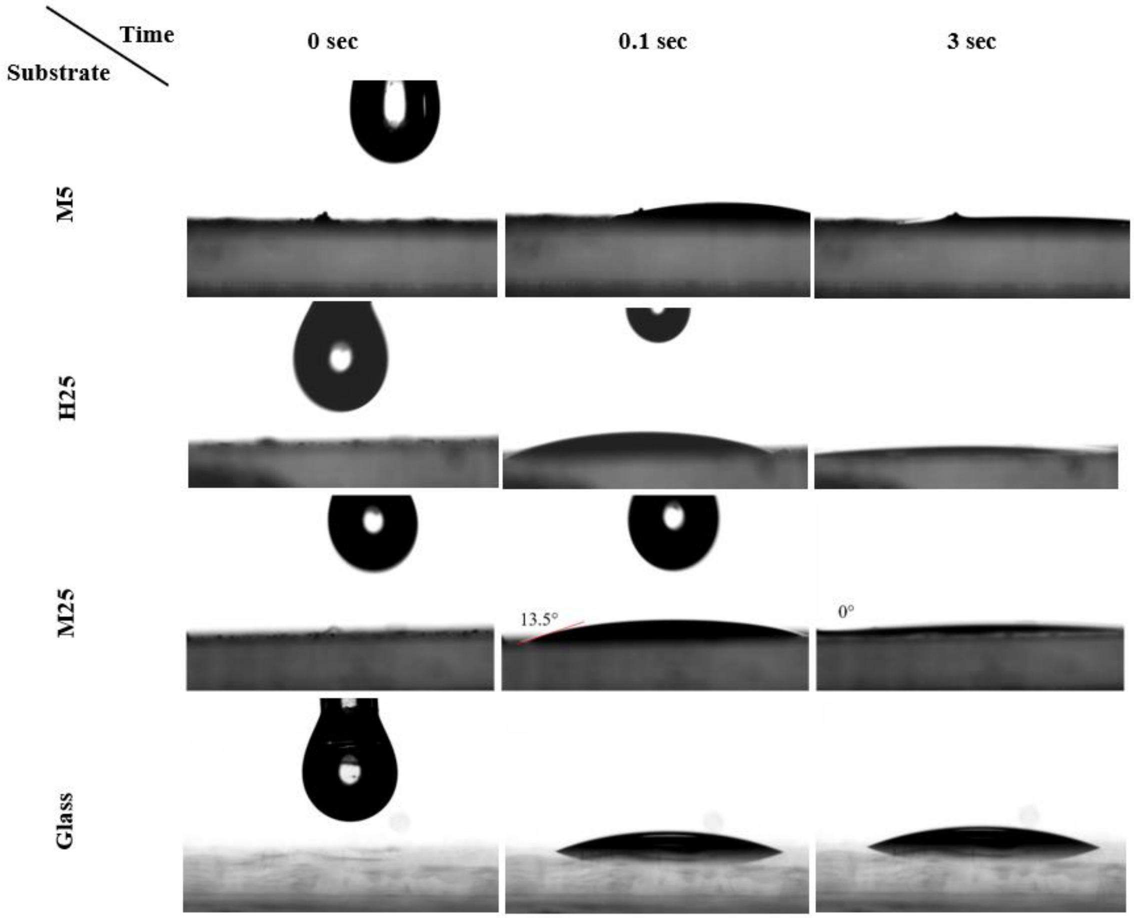

Figure S3. Contact angle measurement of molten paraffin droplet on GNP thin films and glass after 0 sec, 0.1 sec, and 3 sec.

Section S5 Dimensionless Particle Concentration

The dimensionless particle concentration was calculated using the GNP filler dimensions as provided by the manufacturer (Table 2) is presented in Table S2.

Table S3. Theoretical dimensionless particle concentration *c* for composites at different wt% and filler aspect ratio, for the manufacturer provided GNP geometry.

**Filler wt%**	**Dimensionless particle concentration *c***		
	**H25**	**M25**	**M5**
2	16.9	37.3	7.5
5	43.4	95.5	19.1
8	71.9	158.2	31.6
10	91.8	201.9	38.2

As the GNP thickness for M25 is significantly larger compared to the information provided by the manufacturer we assume a thickness of 83 nm for the H25 and M25 filler. For the M5 filler we assume a particle thickness of 40 nm as the exfoliation process will have been different for these particles compared to the larger GNPS. This assumed sheet thickness of 40 nm for the M5 sheets places the percolation threshold slightly above 5 wt% which matched the thermal conductivity increase shown in Figure 5. The resulting dimensionless particle concentration is presented in Table S3.

Table S4. Corrected dimensionless particle concentration *c* for composites at different wt% and filler aspect ratio, with a corrected GNP sheet thickness.

Filler wt%	Dimensionless particle concentration *c*		
	H25	M25	M5
2	3.8	3.7	1.6
5	7.7	7.5	3.2
8	15.3	15.0	6.4
10	19.2	18.7	8.0

Section S6 Experimental Sheet Thickness of the M25 filler

The GNP sheet thickness for M25 provided by the manufacturer is 7 nm however, the average sheet thickness observed in a cross section (Figure S2) prepared by focused ion beam (FIB) cutting under cryogenic conditions and imaged by SEM is 83 ± 7.4 nm.

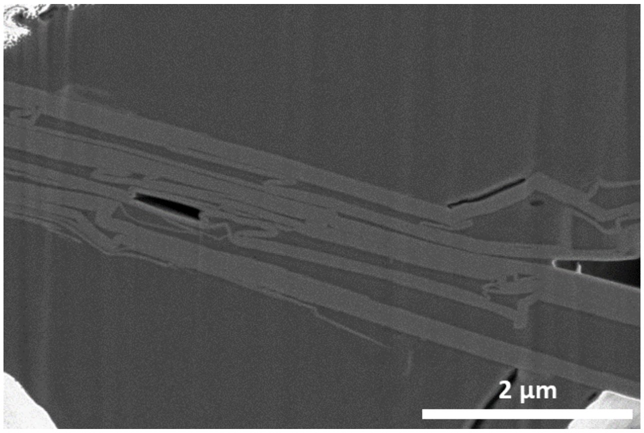

Figure S4. SEM image of cryo FIB cross section of PA-5-M25.
